# The Japanese breast cancer society clinical practice guidelines for breast cancer screening and diagnosis, 2022 edition

**DOI:** 10.1007/s12282-023-01521-x

**Published:** 2023-11-16

**Authors:** Kazunori Kubota, Kazutaka Nakashima, Kazuaki Nakashima, Masako Kataoka, Kenich Inoue, Mariko Goto, Chizuko Kanbayashi, Koichi Hirokaga, Ken Yamaguchi, Akihiko Suzuki

**Affiliations:** 1 Department of Radiology, Dokkyo Medical University Saitama Medical Center, 2-1-50 Minami-koshigaya, Koshigaya, Saitama 343-8555 Japan; 2 The Japanese Breast Cancer Society Clinical Practice Guidelines Breast Cancer Screening and Diagnosis Subcommittee, Tokyo, Japan; 3 Department of General Surgery, Kawasaki Medical School General Medical Center, Okayama, Japan; 4 Division of Breast Imaging and Breast Interventional Radiology, Shizuoka Cancer Center, Shizuoka, Japan; 5 Department of Diagnostic Imaging and Nuclear Medicine, Kyoto University Graduate School of Medicine, Kyoto, Japan; 6 Breast Cancer Center, Shonan Memorial Hospital, Kanagawa, Japan; 7 Department of Radiology, Graduate School of Medical Science, Kyoto Prefectural University of Medicine, Kyoto, Japan; 8 Department of Breast Oncology, Niigata Cancer Center Hospital, Niigata, Japan; 9 Department of Breast Surgery, Hyogo Cancer Center, Hyogo, Japan; 10 Department of Radiology, Faculty of Medicine, Saga University, Saga, Japan; 11 Division of Breast and Endocrine Surgery, Tohoku Medical and Pharmaceutical University, Sendai, Japan

**Keywords:** Japanese breast cancer society, Clinical practice guidelines, Breast cancer screening, Breast cancer surveillance, Breast cancer diagnosis

## Abstract

This article provides updates to readers based on the newly published Japanese Breast Cancer Society Clinical Practice Guidelines for Breast Cancer Screening and Diagnosis, 2022 Edition. These guidelines incorporate the latest evaluation of evidence from studies of diagnostic accuracy. For each clinical question, outcomes for benefits and harms were established, and qualitative or quantitative systematic reviews were conducted. Recommendations were determined through voting by a multidisciplinary group, and guidelines were documented to facilitate shared decision-making among patients and medical professionals. The guidelines address screening, surveillance, and pre- and postoperative diagnosis of breast cancer. In an environment that demands an integrated approach, decisions are needed on how to utilize modalities, such as mammography, ultrasound, MRI, and PET/CT. Additionally, it is vital to understand the appropriate use of new technologies, such as tomosynthesis, elastography, and contrast-enhanced ultrasound, and to consider how best to adapt these methods for individual patients.

## Introduction

The Japanese Breast Cancer Society (JBCS) Clinical Practice Guidelines for Breast Cancer Screening and Diagnosis, 2022 Edition provides consensus statements on current approaches to breast cancer screening and diagnosis. From the 2018 edition [1], the practice guidelines are intended to facilitate shared decision-making on all aspects of breast cancer screening and diagnosis. The guidelines were developed in accordance with the Minds Manual for Guideline Development 2020 ver. 3.0 [2]. Outcomes as specific indicators of benefits and harms were established and evaluated by quantitative or qualitative systematic review. QUADAS-2 (A Revised Tool for the Quality Assessment of Diagnostic Accuracy Studies 2) [3] was used to assess the quality of studies of diagnostic accuracy. Finally, recommendations were determined through discussion and voting at a recommendation decision meeting, including physicians, nurses, and breast cancer survivors. This article summarizes the practice guidelines, including eight clinical questions (CQs) and two background questions (BQs), supported by recommendations and evidence, along with the weight of consensus among the expert panelists and supporting references.

## Practice guidelines for breast cancer screening

### CQ1. Is handheld ultrasound recommended for population-based breast cancer screening?

#### Recommendation

We recommend use of handheld ultrasound as an adjunct to population-based breast cancer screening mammography. [Strength of Recommendation (SoR), 2; Strength of Evidence (SoE), moderate, consensus rate: 94% (45/48)].

We advise against using handheld ultrasound alone for population-based breast cancer screening. [Strength of Recommendation (SoR), 3; Strength of Evidence (SoE), moderate, consensus rate: 94% (40/46)].

#### Justification

The only randomized-controlled trial (RCT) of handheld ultrasound, J-START, found a significant increase in the number of Stage I breast cancers detected (control group, *n* = 48 cases; intervention group, *n* = 93) and a 50% reduction in intermediate-stage cancers (control group, *n* = 35; intervention group, *n* = 18) [4]. This suggests that combined use of ultrasound with mammography may indirectly contribute to a sense of reassurance among examinees. There is concern about an increase in false-positive results, but this can be managed at an acceptable level through appropriate quality control. Therefore, it is weakly recommended to conduct screening using both mammography and ultrasound, provided that proper quality control is ensured. On the other hand, the sensitivity of ultrasonography alone is not superior to that of mammography, and the mortality reduction proven with mammography has not been shown for ultrasound [5, 6]. Therefore, ultrasonography alone is not superior to mammography screening and it is weakly recommended to avoid screening using ultrasound alone.

## Practice guidelines forbreast cancer surveillance

### CQ2. Is contrast-enhanced breast MRI surveillance recommended for BRCA mutation carriers?

#### Recommendation

We recommend use of contrast-enhanced breast MRI surveillance for Japanese BRCA mutation carriers [SoR, 1; SoE, moderate, consensus rate: 80% (39/49)].

#### Justification

A qualitative systematic review was conducted for BRCA pathogenic variant carriers who did and did not undergo MRI-inclusive surveillance [7–31]. The overall survival rate, sensitivity, and false-positive rate indicated favorable results with MRI-inclusive surveillance [7–21]. Regarding the side effects of gadolinium contrast media, nephrogenic systemic fibrosis (NSF) can be managed through renal function evaluation. Long-term follow-up data showed no evidence of clinical symptoms associated with deposition of the contrast agent in the brain [31]. It is recommended that surveillance using contrast-enhanced breast MRI be performed at facilities with specialists with knowledge of this method and in collaboration with facilities that offer MRI-guided biopsy. For asymptomatic BRCA carriers, it is suggested to conduct surveillance at a center that provides genetic counseling and post-screening follow-up.

## Practice guidelines for breast cancer diagnosis

### CQ3. Is breast tomosynthesis as an adjunct to mammography recommended in a diagnostic setting?

#### Recommendation

We recommend breast tomosynthesis as an adjunct to mammography in a diagnostic setting [SoR, 2; SoE, weak, consensus rate: 88% (42/48)].

#### Justification

In a quantitative meta-analysis [32–45], we found pooled sensitivity of 86.9% and specificity of 88.4% for breast cancer diagnosis using tomosynthesis, with a false-positive rate from 0 to 67.6% [32–41]. Compared to mammography, tomosynthesis tended to have higher sensitivity and specificity for breast cancer diagnosis and a lower false-positive rate. With regard to radiation dose, the diagnostic reference level (DRL) for tomosynthesis is 1.5 mGy [46]. Dose reduction can be achieved using this value as a guide in image acquisition. Breast tomosynthesis prior to performance of diagnostic ultrasonography can provide more information and improve the diagnostic accuracy of ultrasonography, compared to prior 2D mammography.

### CQ4. Is breast elastography as an adjunct to B-mode ultrasound recommended in a diagnostic setting?

#### Recommendation

We recommend breast elastography as an adjunct to B-mode ultrasound in a diagnostic setting. [SoR, 1–2; unable to reach an agreement; SoE, moderate, consensus rate: strongly recommended 33% (16/48), weakly recommended 56% (27/48), and weakly not recommended 10% (5/48)].

#### Justification

We performed a quantitative meta-analysis of 7 B-mode ultrasonography articles with 8 qualitative evaluations using strain elastography (SE), 4 semi-quantitative evaluations with SE, and 12 quantitative evaluations using shear-wave elastography (SWE). The results showed that addition of elastography to B-mode ultrasonography resulted in a marked improvement in specificity compared to B-mode ultrasonography alone, whether using SE (qualitative and semi-quantitative) or SWE (quantitative) [47–66]. Biopsy could be avoided in some lesions by adding elastography to B-mode ultrasonography. Because of the slight decrease in sensitivity, the decision to avoid biopsy in practice requires a comprehensive diagnosis that takes into account the elastography results based on differential diagnosis by B-mode ultrasonography.

The recommendation decision meeting did not reach a decision on the level of recommendation, because the opinions were divided between “strongly recommended” and “weakly recommended”.

### CQ5. Is contrast-enhanced ultrasound recommended for distinguishing benign and malignant breast lesions?

#### Recommendation

We recommend use of contrast-enhanced ultrasound for distinguishing benign and malignant breast lesions [SoR, 2; SoE, moderate, consensus rate: 74% (35/47)].

#### Justification

We conducted a meta-analysis using ten papers [67–76] comparing conventional B-mode ultrasonography and contrast-enhanced ultrasonography in differentiating between benign and malignant breast lesions. The pooled sensitivity and specificity of B-mode ultrasonography as a control in the meta-analysis were 89.6% and 60.9%, respectively. Addition of contrast-enhanced ultrasonography to B-mode ultrasonography gave a pooled sensitivity and specificity of 95.1% and 80.9%, respectively, indicating improved diagnostic performance for breast masses. Thus, addition of contrast-enhanced ultrasonography to B-mode ultrasonography improves the diagnostic performance for breast lesions. However, this should be considered on a case-by-case basis, taking into account the additional information provided by contrast-enhanced ultrasonography and the possibility of substituting other examinations.

### BQ1. Is an additional examination required for newly detected lesions on preoperative contrast-enhanced breast MRI?

#### Statement

For MRI-detected lesions that are suspected to be malignant on preoperative contrast-enhanced breast MRI, histological examination should be performed if there is an impact on the surgical procedure. However, the patient should be given multidisciplinary information by the medical provider, including additional examinations such as ultrasound, based on a reliable MRI diagnosis, and the indication for histological examination should reflect the values and wishes of the patient.

#### Justification

We conducted a qualitative systematic review [77–96] and found that false positives occurred in MRI-detected lesions in 44–64.6% of cases. In articles that examined MRI-detected lesions limited to the contralateral breast, false positives for these lesions were reported in 49–86.7% of cases. A 2010 RCT (Comparative Effectiveness of MRI in Breast Cancer (COMICE)) and other studies have found that preoperative MRI increases the number of total mastectomies [97]. Therefore, in principle, the decision on the surgical approach should not be based on preoperative MRI findings alone [98, 99]. Histological diagnosis is recommended for MRI-detected contralateral and ipsilateral lesions that are suspected to be malignant and may affect the surgical approach.

### BQ2. Is whole-body examination with CT, PET, or PET-CT recommended for patients with stage I and II preoperative breast cancer?

#### Statement

Preoperative CT or PET-CT whole-body examination is of low significance in patients with stage I-II breast cancer without signs of distant metastasis. However, systemic examination by CT or PET-CT should be considered in patients who are eligible for preoperative chemotherapy, depending on breast cancer subtype, tumor grade, and patient background.

#### Justification

After a qualitative systematic review [100–108], we decided to define this statement as a BQ, because the previous studies did not provide sufficient evidence to make a decision, and it is unlikely that accumulation of future data will significantly change the outcome. A low prevalence of distant metastases was seen in stage I–II cancers. However, systemic examination should be considered based on the breast cancer subtype, tumor grade, and patient background, which may result in a higher frequency of metastases. In the diagnosis of distant metastasis, FDG-PET/CT has been reported to have high diagnostic performance with sensitivity of 96–100% and specificity of 91–100% [109]. The NCCN guidelines include FDG-PET/CT as an optional procedure for patients with cT2 or higher or cN + (Stage II or higher) when preoperative pharmacologic therapy is considered (since ver. 8.2021) [110]. In addition, some patients may wish to have the examination, and if the benefits and harms are fully explained to the patient and the physician determines that there is a need, it may be appropriate to perform the examination.

## Data Availability

Data sharing is not applicable to this article as no datasets were generated or analyzed during the current study.
