# Correction: The Japanese breast cancer society clinical practice guidelines for breast cancer screening and diagnosis, 2022 edition

**DOI:** 10.1007/s12282-023-01533-7

**Published:** 2023-12-11

**Authors:** Kazunori Kubota, Kazutaka Nakashima, Kazuaki Nakashima, Masako Kataoka, Kenich Inoue, Mariko Goto, Chizuko Kanbayashi, Koichi Hirokaga, Ken Yamaguchi, Yusuke Ohta, Akihiko Suzuki

**Affiliations:** 1 Department of Radiology, Dokkyo Medical University Saitama Medical Center, 2-1-50 Minami-koshigaya, Koshigaya, Saitama 343-8555 Japan; 2 The Japanese Breast Cancer Society Clinical Practice Guidelines Breast Cancer Screening and Diagnosis Subcommittee, Tokyo, Japan; 3 Department of General Surgery, Kawasaki Medical School General Medical Center, Okayama, Japan; 4 Division of Breast Imaging and Breast Interventional Radiology, Shizuoka Cancer Center, Shizuoka, Japan; 5 Department of Diagnostic Imaging and Nuclear Medicine, Kyoto University Graduate School of Medicine, Kyoto, Japan; 6 Breast Cancer Center, Shonan Memorial Hospital, Kanagawa, Japan; 7 Department of Radiology, Graduate School of Medical Science, Kyoto Prefectural University of Medicine, Kyoto, Japan; 8 Department of Breast Oncology, Niigata Cancer Center Hospital, Niigata, Japan; 9 Department of Breast Surgery, Hyogo Cancer Center, Hyogo, Japan; 10 Department of Radiology, Faculty of Medicine, Saga University, Saga, Japan; 11 Division of Breast and Endocrine Surgery, Tohoku Medical and Pharmaceutical University, Sendai, Japan

**Correction: Breast Cancer** 10.1007/s12282-023-01521-x

In this article Yusuke Ohta at affiliations ‘The Japanese Breast Cancer Society Clinical Practice Guidelines Breast Cancer Screening and Diagnosis Subcommittee, Tokyo, Japan’ and ‘Department of General Surgery, Kawasaki Medical School General Medical Center, Okayama, Japan’ was missing from the author list.

